# Management of Dyslipidemia in Women and Men with Coronary Heart Disease: Results from POLASPIRE Study

**DOI:** 10.3390/jcm10122594

**Published:** 2021-06-11

**Authors:** Małgorzata Setny, Piotr Jankowski, Agnieszka Krzykwa, Karol A. Kamiński, Zbigniew Gąsior, Maciej Haberka, Danuta Czarnecka, Andrzej Pająk, Paweł Kozieł, Karolina Szóstak-Janiak, Emilia Sawicka, Zofia Stachurska, Dariusz A. Kosior

**Affiliations:** 1 Clinical Cardiology Center, Central Research Hospital the Ministry of The Interior and Administration, 02-507 Warsaw, Poland; 2 I Department of Cardiology, Interventional Electrocardiology and Hypertension, Institute of Cardiology, Jagiellonian University Medical College, 31-008 Kraków, Poland; piotrjankowski@interia.pl (P.J.); danuta.czarnecka@uj.edu.pl (D.C.); tragez88@wpl.pl (P.K.); 3 Polish Mother’s Memorial Hospital Research Institute, 93-338 Łódź, Poland; 4 Department of Cardiology and Hypertension with the Electrophysiological Lab, Central Research Hospital the Ministry of The Interior and Administration, 02-507 Warsaw, Poland; pawlakagnieszka@interia.pl; 5 Department of Population Medicine and Lifestyle Diseases Prevention, Medical University of Bialystok, 15-089 Białystok, Poland; fizklin@wp.pl (K.A.K.); emiliasawickak@gmail.com (E.S.); zofia.stachurska@umb.edu.pl (Z.S.); 6 Department of Cardiology, Medical University of Silesia, 40-055 Katowice, Poland; zgasior@ptkardio.pl (Z.G.); mhaberka@op.pl (M.H.); karolinaszostak@gmail.com (K.S.-J.); 7 Department of Epidemiology and Population Studies, Institute of Public Health, Jagiellonian University Medical College, 31-066 Kraków, Poland; andrzej.pajak@uj.edu.pl; 8 Cardinal Stefan Wyszyński University, 01-815 Warsaw, Poland

**Keywords:** coronary artery disease, hypercholesterolemia, lipid goal attainment, secondary prevention in women

## Abstract

Cardiovascular diseases (CVDs) are the leading cause of death in Poland. Starting from 1992, a gradual decrease in mortality due to CVDs has been observed, which is less noticeable in women. Following this notion, we assessed sex differences in the implementation of ESC recommendations regarding lipid control and the use of statins as part of secondary CVDs prevention in 1236 patients with acute coronary syndrome or elective coronary revascularization within the last 6–24 months. During hospitalization women had more frequently abnormal TC levels than men (*p* = 0.035), with overall higher TC levels (*p* = 0.009) and lower HDL-C levels (*p* = 0.035). In the oldest group, they also had more frequently elevated LDL-C levels (*p* = 0.033). Similar relationships were found during the follow-up visit. In addition, women less often achieved the secondary lipid therapeutic goal for non-HDL-C (*p* = 0.009). At discharge from hospital women were less frequently prescribed statins (*p* = 0.001), which included high-intensity statins (*p* = 0.002). At the follow-up visit the use of high-intensity statins was still less frequent in women (*p* = 0.02). We conclude that women generally have less optimal lipid profiles than men and are less likely to receive high-intensity statins. There is a need for more organized care focused on the management of risk factors.

## 1. Introduction

CVDs are the leading cause of death in Poland. According to the Statistics Poland Report in 2016, they accounted for 43.3% of all deaths [1]. While in 2016 the death rate due to CVDs per 100,000 population was clearly higher in women than in men (457.2 vs. 415.7) [1], the European Society of Cardiology (ESC) guidelines for secondary prevention of ischemic heart disease are the same for both sexes [2]. For both groups, experts issue the same recommendations for lifestyle changes, treatment of comorbidities, use of cardioprotective drugs and invasive coronary artery disease (CAD) treatment therapies. On the other hand, it is known that CVDs appear in women at an older age than in men [3], which affects the range of practically applicable therapeutic options. Moreover, numerous studies have shown a greater number of complications after coronary revascularization procedures in women [4,5,6].

Since the management of CVDs risk factors, such as hypercholesterolemia, play an extremely important preventive role [7,8] there is a need to recognize sex-related differences in this respect. One of the best studied drugs in CVDs prevention are statins. Their effect on reducing cardiovascular (CV) deaths has been proven in numerous clinical trials [9,10,11,12,13]. Furthermore, high-intensity statins have been shown to be more effective than low-intensity statins in reducing recurrent CV events and mortality [14,15], while maintaining the safety of this therapy in both sexes [16]. Similar observations were noted for the use of combination therapies, i.e., the combination of statins and ezetimibe [17,18], as well as in the use of PCSK9 inhibitors [19,20], which are hardly available due to their price.

The aim of this study was to assess the implementation of the ESC recommendations regarding the achievement of target LDL-C levels and the use of statins as part of secondary CAD prevention in men and women.

## 2. Materials and Methods

This work was carried out on a group of 1236 patients included in the multicenter, cross-sectional POLASPIRE study (Polish Action on Secondary and Primary Prevention by Intervention to Reduce Events) [21], which was an extension of the EUROASPIRE V study (European Action on Secondary and Primary Prevention by Intervention to Reduce Events) [22], coordinated by the ESC. It was based on the analysis of patient data from 14 cardiology departments representing various reference levels, including university clinics and regional hospitals located in four geographical regions of Poland (Kraków, Katowice, Białystok, Warsaw). The study included patients between 18 and 80 years of age who had been hospitalized in the last 6–24 months due to one of the following qualifying incidents: acute coronary syndrome (ACS), regardless of its treatment (i.e., ST-segment elevation myocardial infarction (STEMI), non-ST segment elevation (NSTEMI), unstable coronary artery disease (UA)), having had elective coronary angioplasty (PCI), or elective coronary artery bypass surgery (CABG)). The study consisted of two independent parts carried out in 2017 and 2018. Both parts were performed by centrally trained research personnel. Four regional coordinators were responsible for obtaining approvals from local Bioethics Committees. All participants provided written informed consent.

The first part of the study consisted of the review of the patient’s medical history gathered during hospitalization due to the qualifying incident. Its purpose was to obtain information on the risk factors identified before hospitalization, the results of anthropometric measurements (body weight, height, waist circumference), blood pressure values, the results of biochemical tests such as glucose, HBA1c, creatinine and plasma lipids, as well as the procedures performed during the patient’s hospitalization and the pharmacological treatment recommended on the day of discharge. Patients who met the inclusion criteria for the study were subsequently invited to visit the coordinating centre in their region. During the visit, patients were interviewed using detailed EUROASPIRE V questionnaires [22] which concerned: medical history, education, socioeconomic status, smoking, physical activity, diet, participation in cardiac rehabilitation programs, diabetes training, alcohol consumption, and medications. In addition, during the visit, blood pressure (average of two measurements) and heart rate were assessed, anthropometric measurements such as waist circumference, body weight and height were taken, the concentration of carbon monoxide in the exhaled air was determined, and a blood sample was taken for laboratory tests, such as: lipid profile, glucose, creatinine (GFR was calculated using the MDRD formula) or HbA1c. An oral glucose load test was performed in patients without diagnosed diabetes. In all centres, the measurements were performed using similar tools. A blood sample was collected in the morning after overnight fasting. Total cholesterol (TC), high-density lipoprotein cholesterol (HDL-C) and triglycerides (TG) were analysed in serum and HbA1c in whole blood. Low-density lipoprotein cholesterol (LDL-C) was calculated according to Friedewald’s formula. The level of non-HDL was calculated from the formula: TC minus HDL-C.

The presence of hypertension and dyslipidaemia was assigned based on information from prior diagnosis which was included in medical documentation, or the information card from the initial hospitalization. The presence of diabetes mellitus (DM) was assessed either based on prior diagnosis or current glucose metabolism determined at the follow-up visit after an oral glucose load test, according to standard criteria. Smoking status was evaluated based on the interview taken during the follow-up visit. The presence of obesity was defined as body mass index (BMI) ≥ 30 kg/m^2^, based on measurements taken during follow-up.

Because the study was an extension of the EUROASPIRE V study, in which the aims included the assessment of the implementation of the 2016 ESC guidelines for the prevention of cardiovascular disease in clinical practice, our adopted definitions of risk factors and target treatment goals were also based on these guidelines [2]. Accordingly, the following goals were defined: controlled diabetes HbA1c < 7.0%; the lipid primary goal in the treatment of dyslipidaemia, i.e. LDL-C level < 70 mg/dL or a reduction of at least 50% if the baseline LDL-C level was between 70 and 135 mg/dL; the lipid secondary goal in the treatment of dyslipidaemia, i.e. non-HDL-C < 100 mg/dL; normal systolic blood pressure (SBP) < 140 mmHg and normal diastolic blood pressure (DBP) < 90 mmHg for all except diabetic patients where the target DPB was <85 mmHg; normal BMI 20.0–25.0 kg/m^2^; normal waist circumference (WC): women < 80 cm, men < 94 cm; not smoking, and regular physical activity corresponding to moderate-intensity exercise ≥ 150-min/week or vigorous exercise ≥ 75-min/week.

The following lipid levels were considered normal: TC < 190 mg/dL, TG < 150 mg/dL, HDL-C ≥ 40 mg/dL in men and HDL-C ≥ 45 mg/dL in women. Because most patients were at very high CV risk already prior to hospitalization, we adopted a baseline LDL-C < 70 mg/dL as a normal value. As a high-intensity statin therapy, we considered the use of atorvastatin ≥ 40 mg or rosuvastatin ≥ 20 mg.

Statistical analysis. In the case of descriptive statistics, the significance of results was inferred based on the two-tailed Student’s t-test for normally distributed variables (*p*-values > 0.05 in the Shapiro–Wilk tests) or otherwise the Wilcoxon test. In case of categorical variables, the Chi-Square statistic was used. Multivariate logistic regression was used to assess the correlation of observables with dependent variables. All analyses were performed with the “stats” package of the R program, version 3.6.3.

## 3. Results

### 3.1. Clinical Characteristics of the Study Group

The study group included 1236 patients, 354 women (29%) and 882 men (71%). The mean age of women was 66.3 years (SD: 8.6 years), and the mean age of men was 64.1 years (SD: 8.3 years). More than 20% of patients smoked, over 40% had diabetes and obesity, over 80% dyslipidaemia and over 90% hypertension, respectively. A comparison of the basic characteristics of men and women is presented in Table 1.

Women were statistically significantly older than men, on average by about 2 years (*p* < 0.001). In the group under the age of 60, there was a visibly higher prevalence of men, and in the group of over 70 years, of women (*p* < 0.001). Albeit a small in percentage, statistically significant differences were observed in the distribution of the type of qualifying incident (*p* = 0.007). Patients with ACS accounted for 64% and 58% of the female and male groups, respectively, with acute myocardial infarction occurring in 39% of women and 39% of men. It is notable that in women, UA and NSTEMI were more frequent than in men, while STEMI was more frequent in men. Men also underwent elective PCI and CABG more often. Women were statistically obese more often than men (*p* = 0.03).

### 3.2. Lipid Profile at Hospitalization

The information on lipid levels was obtained from medical records from the time of hospitalization, and was available for 993, 1003, 999, and 993 cases for LDL-C, TC, HDL-C, and TG, respectively. This accounted for 80 ± 1% of all included patients, equal for both sexes.

Since the group under study generally included patients with a high CV risk, of whom 43%, equal in men and women, were taking statins prior to hospitalization (14% of patients declared no use of statins, while in another 43% the information was missing or uncertain), we adopted the LDL-C norm < 70 mg/dL. Given this strict condition, target LDL-C levels evaluated during hospitalization were not met by roughly 80% of patients in both sex groups; however, a slight tendency for improvement with age was observed in men, but not women, leading to statistically significant difference in the oldest age group, where elevated LDL-C was present in 67% men and 80% women (*p* = 0.033). Other components of the lipid profile also turned out to be more poorly controlled in women than in men (Table 2). Similarly, as in the case of LDL-C, a gradual decrease in the frequency of abnormal TC levels with age was observed only in men, resulting in 19% of men with elevated TC compared to 35% of women in the ≥ 70 age group (*p* = 0.004). In addition, in women, a significantly more frequent occurrence of reduced HDL-C levels was observed in comparison with men (*p* = 0.035), especially in the youngest age group, i.e., <60 years old (*p* = 0.006).

### 3.3. Lipid Profile at Interview

Of 1236 patients enrolled in the study, a group of 1025, including 289 (28%) women and 736 (72%) men, attended the follow-up visit and had lipid level testing. The trends in lipid profiles were generally similar to those observed during hospitalization (Table 3). Namely, increased levels of TC were significantly more frequent in women than in men, especially in the group of women ≥ 70 years of age, and decreased levels of HDL-C were found in the youngest group. Above normal LDL-C levels were also recorded more frequently in women (68% W vs. 60% M), and the difference was particularly pronounced in the oldest age group (67% W vs. 53% M, *p* = 0.037). Similar observations were noted for non-HDL-C. Elevated levels were found significantly more often in women (54% W vs. 45% M, *p* = 0.009), especially in the group over 70 years of age (53% W vs. 33% M, *p* = 0.0017).

### 3.4. The Achievement of Lipid Treatment Goals

LDL-C levels from the time of hospitalization as well as from the follow-up visit were available in 813 patients, including 227 (28%) women and 586 (72%) men, and non-HDL-C levels for 1025 patients, of whom 291 (28%) were women and 734 (72%) were men.

Overall, there were no statistically significant differences between the sexes in the achievement of the therapeutic goal, i.e., LDL-C < 70 mg/dL or a ≥ 50% reduction of the baseline LDL-C level if it was between 70 and 135 mg/dL (Table 4). In men, with age, there was a tendency to an increase in the frequency of maintaining the recommended LDL-C level. In women, a particularly low success rate in achieving the target LDL-C was observed in the youngest group. Although it showed a tendency to increase with age, notably in the 60–70 years age group, it was still reached somewhat less frequently than in men across all age groups, though statistical significance was not reached due to the limited group size. We note that 39% of patients included in an LDL-C baseline level assessment were taking statins, which likely contributed to its generally low value.

The secondary therapeutic lipid goal, namely non-HDL-C < 100 mg/dL, was met statistically significantly less often in women than in men. Overall, 46% of women and 55% of men (*p* = 0.009) achieved the goal. The difference between sexes was most apparent in the oldest age group, i.e., ≥70 years of age (Table 4).

According to the logistic regression model based on gender, age and participance in the rehabilitation program, male gender (*p* = 0.048) and advanced age (*p* = 0.027) were significantly associated with the attainment of the recommended LDL-C level.

### 3.5. LDL-C and Non-HDL-C Profiles, and the Lipid Goals Achievement According to 2019 ESC Guidelines on Dyslipidaemias

The most recent ESC guidelines announced in 2019 introduced even stricter goals for the management of dyslipidemias. In very high CV risk patients at least a 50% reduction from baseline LDL-C levels and a target concentration < 55 mg/dL are now recommended, whereas the non-HDL-C goal has been lowered to <85 mg/dL. If such criteria were applied to our study group patients (Table 5), the LDL-C goal would be achieved by only 7% of women and 8% of men (*p* = 0.96). In turn, the non-HDL-C goal would be met by 23% of women and 35% of men (*p* < 0.001). Target LDL-C levels evaluated during hospitalization would not be met by roughly 90% of patients in both sex groups. Above normal LDL-C levels at follow-up would be found in 85% of women and 80% of men (*p* = 0.12).

### 3.6. Lipid Lowering Therapies

After a qualifying incident, both genders were equally educated about hypercholesterolemia management, with 25% of women and 27% of men (*p* = 0.58) reporting that the educator was a hospital doctor, a family doctor in 43% of women and 48% of men (*p* = 0.19), and a cardiologist in 57% of women and 61% of men (*p* = 0.23).

Women were more likely than men to report that they reduced their dietary fat intake (79% vs. 68%; *p* = 0.001); otherwise, there were no gender differences in the frequency of lifestyle changes to combat hypercholesterolemia. The change in the type of consumed fat was reported by 66% of women and 59% of men (*p* = 0.054), an increase in dietary vegetables and fruits content by 75% of women and 70% of men (*p* = 0.11), inclusion of more fish by 43% of women and 45% of men (*p* = 0.62), and fatty fish by 33% of women and 36% of men (*p* = 0.42), and finally, regular consumption of foods enriched with vegetable fats by 44% of women and 41% of men (*p* = 0.44).

At discharge from hospital due to a qualifying incident, women were prescribed statins less frequently (*p* = 0.001), which was particularly significant in the group of ≥70 years of age (*p* = 0.011). At the time of discharge, women were also less likely (*p* = 0.002) to receive a high-intensity statin therapy (i.e., atorvastatin ≥ 40 mg or rosuvastatin ≥ 20 mg). The observed trend was particularly evident in the group of women between 60 and 70 years of age, as compared to men of the same age (*p* = 0.005) (Table 6). Concerning the type of qualifying incident, the disproportion was most apparent in the case of a high intensity statin prescription in patients with STEMI (72% vs. 90%; *p* = 0.004) (Table 7). The differences in general statin usage between both sex groups were less pronounced during the follow-up visit. Overall, statins were taken by 87% of women and 90% of men (*p* = 0.17), respectively. High intensity statins were still used less frequently in women than in men (53% vs. 61%; *p* = 0.02).

There were no statistically significant differences between the sexes in terms of the reasons for discontinuation or reduction of the statin dose (Table 8). The most frequently reported reason for therapy discontinuation for women was drug intolerance, while in men it was the doctor’s recommendation. Such a recommendation was also the dominant cause for statin dose reduction in both sexes. Finally, the most prevalent symptom of drug intolerance in both sexes was muscle pain.

It is apparent that the sustainability of statin therapy highly depends on the kind of medical care received by the patient. At the follow-up visit, among patients who declared to remain under the care of any medical doctor (n = 1003), statins were taken significantly more frequently by those who visited a cardiologist compared to those who did not consult such a specialist (91% of n = 868 vs. 81% of n = 135; *p* < 0.001). Similar dependence was observed in the case of strong statins (60% vs. 50%; *p* = 0.03). Other proven options of lipid lowering therapy, such as the use of ezetimibe are generally poorly implemented even by specialists, as is evidenced by their introduction to only 3% of patients remaining under the care of a cardiologist.

## 4. Discussion

Lipid disorders are a major risk factor of CVDs, whose high prevalence in the general population has been confirmed in a number of reports [23,24]. For instance, in the WOBASZ II study concerning the Polish population [25], hypercholesterolemia was identified in 70% of men and 64% of women. Accordingly, proper management of the lipid profile is of high clinical importance. Our analysis indicates that the lipid profile is generally less well controlled in women than in men, with a significantly higher incidence of above normal LDL-C and TC levels, especially in the oldest age group, and below normal HDL-C level in the youngest group. Moreover, a large percentage of patients with high CV risk who remained under medical care, did not achieve the recommended therapeutic goals with respect to lipid levels. Again, these goals are generally less frequently met among women than men: recommended LDL-C was observed in only 20% of women compared to 25% of men, and non-HDL-C in 46% of women compared to 55% of men.

The above observations based on the Polish population coincide with the results of subsequent editions of the EUROASPIRE studies [24,26,27], concerning the implementation of the new guidelines for CVD prevention in clinical practice in Europe, as well as with other reports [28]. For example, in the study by Zhao et al., which enrolled 10,112 patients, 29% of whom were women, women were less likely to achieve the target level of TC and LDL-C [29].

Lower LDL-C levels are associated with lower rates of major coronary events [30], and the reduction of CV risk is proportional to the absolute reduction in LDL-C levels [31]. While this effect is not specific to any particular kind of therapy [32], a major role in lipid management is played by statins which are widely available and their effectiveness in lowering of LDL-C levels has been proven in numerous clinical studies. Indeed, a number of reports have shown that the use of statins and the reduction of LDL-C levels translate into a decrease in mortality and morbidity due to CVDs [9,10]. These observations were included in the Third Declaration of Sopot from 2018 [33] and in the ESC/EAS guidelines for the management of dyslipidemias from 2019 [34], in which the LDL-C goal was lowered in all CV risk categories. Obviously, if interpreted according to these new guidelines, the results obtained in our study would indicate an even higher prevalence of dyslipidemia and even more frequent failure to achieve target lipid levels in both genders. It is of note that while in both sexes the very high baseline rates of above normal lipid levels recorded at hospitalization are somewhat reduced at the follow-up visit, it is apparent, both in the case of LDL-C and non-HDL-C in particular, that this reduction is more efficient in men than in women. Altogether, the LDL-C goal would be achieved by only 7% of women and 8% of men, and the non-HDL-C goal would be met by 23% women and 35% men. It should be kept in mind that lipid lowering therapy assessed by the POLASPIRE study followed 2016 guidelines and was aimed at the goals defined thereof, and hence the above numbers do not fairly represent success rate in lipid management according to current recommendations; nevertheless, they still demonstrate differences between sexes to the disadvantage of women.

The apparent trend in imposing more ambitious lipid management goals further signifies the importance of correctly executed lipid lowering therapy. According to our results, at discharge from hospital, women were prescribed statins less frequently than men. They were also taking high-intensity statins statistically significantly less frequently, and these disproportions were visible in all analyzed age subgroups. Of particular note was the significantly less frequent prescription of high intensity statins to women after STEMI. These observations are consistent with previous reports. In the meta-analysis by Zhao et al. [35], which included more than two million patients at high CV risk or with already confirmed CVD, women were less likely to be prescribed statins, aspirin, and ACE-inhibitors. In the analyses by Peters et al. [36] women who were discharged after hospitalization because of myocardial infarction were less likely to receive prescriptions for high-intensity statins (i.e. atorvastatin in dose of 40–80 mg and rosuvastatin in dose of 20–40 mg). Similarly, in the PALM study [37], women more often reported that they had never been recommended such therapy. In the study by Nanna et al. [38] women also received it less frequently, but also often refused such treatment or discontinued it themselves. There are several possible reasons why women may be prescribed statins less frequently. One of them is the belief that women suffer less from CVDs and therefore do not require as intensive care as men. Another cause is the fear of doctors and patients about possible side effects of drugs, reported more often by women [39,40], which may influence decisions about using these drugs in lower doses or not including them altogether.

It is worth emphasizing that according to our observations patients who remained under the care of a cardiologist were more likely to receive statins in general, as well as high intensity statins. It also stems from the patients’ reports, that among physicians involved in their treatment, it was the cardiologist who most often educated them about the treatment of hypercholesterolemia. Unfortunately, however, many of them did not introduce the recommended lifestyle changes.

The study has several limitations. It included patients after acute coronary syndrome and after elective revascularization treatment in the last 6–24 months, so the assessment was limited to a selected group of patients with CAD. Baseline lipid profile values were based on medical documentation and were unknown in approximately 20% of patients. In addition, data regarding the status of hypolipemic treatment prior to hospitalization were either not available or uncertain in 43% of cases. There were 17% of patients invited to participate in the study who did not attend the follow-up visit. Considering that these individuals may have had less interest in controlling CV risk factors, their adherence to recommendations may also have been poorer, which in turn implies some degree of bias within the remaining group towards overall better compliance. Finally, the study did not cover all regions of Poland.

In conclusion, the study showed that a high percentage of patients, especially women, after acute coronary syndrome or elective coronary revascularization procedures, did not achieve the recommended goals in the treatment of dyslipidemia. This leaves a lot of room for improvement and indicates the crucial importance of organized care for high CVD risk patients. There is also an apparent need for training programs, both for patients and doctors, to promote the opportunities for CV risk reduction in this patient group.

## Figures and Tables

**Table 1:** Clinical characteristics of the groups of women and men.

Parameter	Women n (%)	Men n (%)	*p*
Patients n (%)	354 (29)	882 (71)	
Mean age (SD)	66.3 (9)	64.1 (8)	<0.001
Age groups n (%)			<0.001
<60	71 (20)	263 (30)	
60–70	157 (45)	403 (46)	
≥70	125 (35)	214 (24)	
Incident n (%)			0.007
elective CABG	8 (2)	46 (5)	
elective PCI	120 (34)	323 (37)	
STEMI	46 (13)	150 (17)	
NSTEMI	91 (26)	194 (22)	
UA	89 (25)	169 (19)	
Accompanying condition n (%)			
Hypertension	313 (95)	722 (93)	0.21
Diabetes	120 (41)	286 (39)	0.54
Dyslipidemia	234 (81)	599 (81)	0.95
Active smoking	38 (21)	132 (23)	0.69
Obesity	141 (48)	293 (40)	0.034

CABG, coronary artery bypass surgery; NSTEMI, non-ST segment elevation myocardial infarction; PCI, coronary angioplasty; STEMI, ST-segment elevation myocardial infarction; UA, unstable coronary artery disease.

**Table 2:** Lipid profile according to gender and age at hospitalization.

Age Group	Women	Men	*p*	Women n (%)	Men n (%)	*p*
	LDL-C mg/dL: mean (SD)			LDL-C ≥ 70 mg/dL		
All	107 (43)	104 (45)	0.22	224 (79)	542 (76)	0.32
<60	117 (49)	116 (51)	0.96	46 (84)	167 (81)	0.86
60–70	107 (45)	103 (43)	0.42	95 (77)	256 (78)	0.99
≥70	102 (37)	93 (37)	0.026	83 (80)	119 (67)	0.033
	TC mg/dL: mean (SD)			TC ≥ 190 mg/dL		
All	177 (48)	169 (49)	0.009	104 (36)	211 (29)	0.035
<60	188 (53)	182 (54)	0.58	23 (41)	81 (39)	0.89
60–70	176 (49)	168 (48)	0.10	44 (35)	96 (29)	0.20
≥70	172 (41)	155 (42)	<0.001	37 (35)	34 (19)	0.004
	HDL-C mg/dL: mean (SD)			HDL-C < 40 mg/dL (M) <45 mg/dL (W)		
All	52 (17)	47 (16)	<0.001	125 (44)	260 (36)	0.035
<60	49 (16)	46 (14)	0.23	33 (59)	77 (38)	0.006
60–70	52 (17)	46 (17)	<0.001	51 (41)	130 (39)	0.78
≥70	54 (17)	47 (14)	<0.001	41 (39)	53 (30)	0.15
	TG mg/dL: mean (SD)			TG ≥ 150 mg/dL		
All	134 (68)	141 (103)	0.77	86 (30)	224 (32)	0.81
<60	153 (89)	151 (91)	0.85	21 (38)	82 (40)	0.85
60–70	132 (59)	147 (119)	0.90	37 (30)	109 (33)	0.68
≥70	127 (64)	117 (79)	0.024	28 (27)	33 (19)	0.15
	non-HDL-C mg/dL: mean (SD)			non-HDL-C ≥ 100 mg/dL		
All	124 (46)	123 (49)	0.31	198 (69)	457 (64)	0.12
<60	138 (51)	136 (54)	0.86	43 (77)	150 (73)	0.70
60–70	123 (49)	122 (48)	0.65	85 (69)	215 (65)	0.51
≥70	118 (38)	108 (41)	0.008	70 (67)	92 (52)	0.022

HDL-C, high-density lipoprotein cholesterol; LDL-C, low-density lipoprotein cholesterol; non-HDL-C, non-high-density lipoprotein cholesterol; TC, total cholesterol; TG, triglycerides.

**Table 3:** Lipid profile according to gender and age at interview.

Age Group	Women	Men	*p*	Women n (%)	Men n (%)	*p*
	LDL-C mg/dL: mean (SD)			LDL-C ≥ 70 mg/dL		
All	90 (40)	85 (43)	0.026	197 (68)	444 (60)	0.037
<60	102 (55)	90 (38)	0.16	44 (77)	136 (65)	0.11
60–70	85 (36)	85 (45)	0.80	85 (64)	210 (62)	0.64
≥70	89 (35)	78 (44)	0.002	68 (67)	98 (53)	0.037
	TC mg/dL: mean (SD)			TC ≥ 190 mg/dL		
All	170 (45)	153 (41)	<0.001	72 (25)	121 (16)	0.003
<60	178 (60)	161 (46)	0.02	13 (23)	47 (22)	1
60–70	164 (40)	153 (41)	0.002	33 (25)	53 (16)	0.024
≥70	172 (42)	144 (35)	<0.001	26 (25)	21 (11)	0.003
	HDL-C mg/dL: mean (SD)			HDL-C < 40 mg/dL (M) < 45 mg/dL (W)		
All	58 (16)	50 (21)	<0.001	86 (30)	189 (26)	0.24
<60	52 (15)	48 (13)	0.056	29 (51)	63 (30)	0.006
60–70	57 (14)	50 (27)	<0.001	38 (29)	91 (27)	0.73
≥70	62 (16)	51 (14)	<0.001	19 (19)	35 (19)	1
	TG mg/dL: mean (SD)			TG ≥ 150 mg/dL		
All	133 (65)	130 (94)	0.003	81 (28)	200 (27)	0.91
<60	142 (58)	146 (95)	0.56	19 (33)	78 (37)	0.68
60–70	135 (61)	135 (108)	0.05	45 (34)	98 (29)	0.30
≥70	126 (74)	104 (48)	<0.001	17 (17)	24 (13)	0.50
	non-HDL-C mg/dL: mean (SD)			non-HDL-C ≥ 100 mg/dL		
All	112 (45)	103 (40)	0.003	158 (54)	331 (45)	0.009
<60	126 (59)	113 (44)	0.14	39 (68)	108 (52)	0.035
60–70	107 (39)	103 (40)	0.30	65 (49)	162 (48)	0.81
≥70	111 (42)	92 (31)	<0.001	54 (53)	61 (33)	0.0017

HDL-C, high-density lipoprotein cholesterol; LDL-C, low-density lipoprotein cholesterol non-HDL-C, non-high-density lipoprotein cholesterol; TC, total cholesterol; TG, triglycerides.

**Table 4:** The achievement of target LDL-C level and non-HDL-C in age groups in women and men during the follow-up visit.

	Women n (%)	Men n (%)	*p*
LDL-C goal in age groups			
All	45 (20)	147 (25)	0.13
<60	5 (10)	33 (19)	0.24
60–70	24 (24)	68 (25)	0.89
≥70	16 (21)	46 (33)	0.08
Non-HDL goal in age groups			
All	133 (46)	403 (55)	0.009
<60	18 (32)	101 (48)	0.035
60–70	67 (51)	179 (52)	0.81
≥70	48 (47)	123 (67)	0.0017

LDL-C, low-density lipoprotein cholesterol; non-HDL-C, non-high-density lipoprotein cholesterol.

**Table 5:** LDL-C and non-HDL-C profiles and the lipid goals achievement according to 2019 ESC guidelines on dyslipidaemias.

	Women n (%)	Men n (%)	*p*
Hospitalization			
LDL-C ≥ 55 mg/dL	260 (92)	636 (89)	0.23
non-HDL-C ≥ 85 mg/dL	235 (82)	551 (77)	0.08
Interview			
LDL-C ≥ 55 mg/dL	246 (85)	588 (80)	0.12
non-HDL-C ≥ 85 mg/dL	223 (77)	476 (65)	<0.001
Lipid goal attainment			
LDL-C	17 (7)	46 (8)	0.95
non-HDL-C	68 (23)	258 (35)	<0.001

LDL-C, low-density lipoprotein cholesterol; non-HDL-C, non-high-density lipoprotein cholesterol.

**Table 6:** Statins prescription and the use of statins in different age groups in men and women on discharge from hospital.

	Women n (%)	Men n (%)	*p*
Statins prescription			
Any statins			
All	321 (91)	843 (96)	0.001
<60	58 (89)	233 (95)	0.2
60–70	146 (93)	388 (96)	0.2
≥70	117 (89)	222 (96)	0.011
High intensity statins			
All	217 (61)	622 (71)	0.002
<60	47 (72)	184 (75)	0.8
60–70	96 (61)	298 (74)	0.005
≥70	74 (56)	140 (61)	0.5
Use of statins			
Any statins			
All	252 (87)	665 (90)	0.17
<60	46 (84)	190 (91)	0.19
60–70	114 (87)	312 (90)	0.36
≥70	92 (89)	163 (90)	1
High intensity statins			
All	152 (53)	448 (61)	0.02
<60	32 (58)	145 (69)	0.16
60–70	72 (55)	208 (60)	0.34
≥70	48 (47)	95 (52)	0.43

**Table 7:** Statins prescription and the use of statins in men and women depending on the type of qualifying incident.

Incident	Women n (%)	Men n (%)	*p*
	Any statins prescription		
elective CABG	7 (88)	45 (98)	0.68
elective PCI	115 (96)	313 (97)	0.79
STEMI	40 (87)	144 (96)	0.06
NTEMI	80 (88)	182 (94)	0.14
UA	77 (87)	157 (93)	0.15
	High intensity statins prescription		
elective CABG	4 (50)	29 (63)	0.76
elective PCI	75 (63)	228 (71)	0.13
STEMI	33 (72)	135 (90)	0.004
NSTEMI	61 (67)	151 (78)	0.07
UA	44 (49)	79 (47)	0.78
	Use of any statins		
elective CABG	7 (100)	32 (89)	0.83
elective PCI	88 (90)	265 (94)	0.25
STEMI	32 (89)	110 (87)	0.94
NSTEMI	63 (88)	134 (89)	0.86
UA	62 (82)	122 (87)	0.44
	Use of high intensity statins		
elective CABG	3 (43)	20 (56)	0.84
elective PCI	50 (51)	185 (66)	0.015
STEMI	23 (64)	91 (72)	0.49
NSTEMI	44 (61)	93 (62)	0.98
UA	31 (41)	58 (41)	0.92

CABG, coronary artery bypass surgery; NSTEMI, non-ST segment elevation myocardial infarction; PCI, coronary angioplasty; STEMI, ST-segment elevation myocardial infarction; UA, unstable coronary artery disease.

**Table 8:** Reasons for discontinuation of statin therapy and statin dose reduction. P value in all cases was insignificant due to the small number of cases.

	Women n (%)	Men n (%)
Reason for interruption		
Intolerance	5 (42)	8 (21)
Patient’s refusal	3 (25)	10 (26)
Doctor’s recommendation	4 (33)	14 (36)
Others	0 (0)	2 (5)
Uncertain/I do not know	0 (0)	5 (13)
Reason for dose reduction		
Intolerance	3 (21)	6 (12)
Doctor’s recommendation	7 (50)	31 (62)
Other	1 (7)	2 (4)
Uncertain/I do not know	3 (21)	11 (22)

## Data Availability

Data available on request from corresponding authors.

## References

[1] (2016). Statistics Poland Report 2016.

[2] Piepoli M.F., Hoes A.W., Agewall S., Albus C., Brotons C., Catapano A.L., Cooney M., Corrà U., Cosyns B., Deaton C. (2016). 2016 European Guidelines on cardiovascular disease prevention in clinical practice: The Sixth Joint Task Force of the European Society of Cardiology and Other Societies on Cardiovascular Disease Prevention in Clinical Practice constituted by representati. Eur. Heart J..

[3] Keteepe-Arachi T., Sharma S. (2017). Cardiovascular Disease in Women: Understanding Symptoms and Risk Factors. Eur. Cardiol. Rev..

[4] Potts J., Sirker A., Martinez S.C., Gulati M., Alasnag M., Rashid M., Kwok C.S., Ensor J., Burke D.L., Riley R.D. (2018). Persistent sex disparities in clinical outcomes with percutaneous coronary intervention: Insights from 6.6 million PCI procedures in the United States. PLOS ONE.

[5] Lam L., Ahn H.J., Okajima K., Schoenman K., Seto T.B., Shohet R.V., Miyamura J., Sentell T.L., Nakagawa K. (2019). Gender Differences in the Rate of 30-Day Readmissions after Percutaneous Coronary Intervention for Acute Coronary Syndrome. Women’s Heal. Issues.

[6] Iantorno M., Torguson R., Kolm P., Gajanana D., Suddath W.O., Rogers T., Bernardo N.L., Ben-Dor I., Gai J., Satler L.F. (2019). Relation of Sex and Race to Outcomes in Patients Undergoing Percutaneous Intervention With Drug-Eluting Stents. Am. J. Cardiol..

[7] Multiple Risk Factor Intervention Trial Research Group (1986). Coronary heart disease death, nonfatal acute myocardial infarction and other clinical outcomes in the multiple risk factor intervention trial. Am. J. Cardiol..

[8] Yusuf S., Hawken S., Ôunpuu S., Dans T., Avezum A., Lanas F., McQueen M., Budaj A., Pais P., Varigos J. (2004). Effect of potentially modifiable risk factors associated with myocardial infarction in 52 countries (the INTERHEART study): Case-control study. Lancet.

[9] Scandinavian Simvastatin Survival Study Group (1994). Randomised trial of cholesterol lowering in 4444 patients with coronary heart disease: The Scandinavian Simvastatin Survival Study (4S). Lancet.

[10] Heart Protection Study Collaborative Group (2002). MRC/BHF Heart Protection Study of cholesterol lowering with simvastatin in 20,536 high-risk individuals: A randomised place-bocontrolled trial. Lancet.

[11] Simes R.J., Marschner I.C., Hunt D., Colquhoun D., Sullivan D., Stewart R.A., Hague W., Keech A., Thompson P., White H. (2002). Relationship Between Lipid Levels and Clinical Outcomes in the Long-Term Intervention With Pravastatin in Ischemic Disease (LIPID) Trial. Circulation.

[12] Kinlay S., Schwartz G.G., Olsson A.G., Rifai N., Sasiela W.J., Szarek M., Ganz P., Libby P. (2004). Effect of Atorvastatin on Risk of Recurrent Cardiovascular Events After an Acute Coronary Syndrome Associated With High Soluble CD40 Ligand in the Myocardial Ischemia Reduction with Aggressive Cholesterol Lowering (MIRACL) Study. Circulation.

[13] Rouleau J. (2005). Improved outcome after acute coronary syndromes with an intensive versus standard lipid-lowering regimen: Results from the Pravastatin or Atorvastatin Evaluation and Infection Therapy–Thrombolysis in Myocardial Infarction 22 (PROVE IT–TIMI 22) trial. Am. J. Med..

[14] Cannon C.P., Steinberg B.A., Murphy S.A., Mega J.L., Braunwald E. (2006). Meta-Analysis of Cardiovascular Outcomes Trials Comparing Intensive Versus Moderate Statin Therapy. J. Am. Coll. Cardiol..

[15] Rodriguez F., Maron D.J., Knowles J.W., Virani S.S., Lin S., Heidenreich P.A. (2017). Association Between Intensity of Statin Therapy and Mortality in Patients With Atherosclerotic Cardiovascular Disease. JAMA Cardiol..

[16] Fulcher J., O’Connell R., Voysey M., Emberson J., Blackwell L., Mihaylova B., Simes J., Collins R., Kirby A., Colhoun H. (2015). Efficacy and safety of LDL-lowering therapy among men and women: meta-analysis of individual data from 174 000 participants in 27 randomised trials. Lancet.

[17] Baigent C., Landray M., Reith C., Emberson J., Wheeler D.C., Tomson C., Wanner C., Krane V., Cass A., Craig J. (2011). The effects of lowering LDL cholesterol with simvastatin plus ezetimibe in patients with chronic kidney disease (Study of Heart and Renal Protection): A randomised placebo-controlled trial. Lancet.

[18] Cannon C.P., Blazing M.A., Giugliano R.P., McCagg A., White J.A., Théroux P., Darius H., Lewis B.S., Ophuis T.O., Jukema J.W. (2015). Ezetimibe Added to Statin Therapy after Acute Coronary Syndromes. N. Engl. J. Med..

[19] Sabatine M.S., Giugliano R.P., Keech A.C., Honarpour N., Wiviott S.D., Murphy S.A., Kuder J.F., Wang H., Liu T., Wasserman S.M. (2017). Evolocumab and Clinical Outcomes in Patients with Cardiovascular Disease. N. Engl. J. Med..

[20] Schwartz G.G., Steg P.G., Szarek M., Bhatt D.L., Bittner V.A., Diaz R., Edelberg J.M., Goodman S.G., Hanotin C., Harrington R.A. (2018). Alirocumab and Cardiovascular Outcomes after Acute Coronary Syndrome. N. Engl. J. Med..

[21] Jankowski P., Kosior D.A., Sowa P., Szóstak-Janiak K., Kozieł P., Krzykwa A., Sawicka E., Haberka M., Setny M., Kamiński K. (2020). Secondary prevention of coronary artery disease in Poland. Results from the POLASPIRE survey. Cardiol. J..

[22] Kotseva K., De Backer G., De Bacquer D., Rydén L., Hoes A., Grobbee D., Maggioni A., Marques-Vidal P., Jennings C., Abreu A. (2019). Lifestyle and impact on cardiovascular risk factor control in coronary patients across 27 countries: Results from the European Society of Cardiology ESC-EORP EUROASPIRE V registry. Eur. J. Prev. Cardiol..

[23] Zdrojewski T., Solnica B., Cybulska B., Bandosz P., Rutkowski M., Stokwiszewski J., Gaciong Z., Banach M., Wojtyniak B., Pencina M. (2016). Prevalence of lipid abnormalities in Poland. The NATPOL 2011 survey. Kardiologia Polska.

[24] De Backer G., Jankowski P., Kotseva K., Mirrakhimov E., Reiner Ž., Rydén L., Tokgözoğlu L., Wood D., de Bacquer D. (2019). Management of dyslipidaemia in patients with coronary heart disease: Results from the ESC-EORP EUROASPIRE V survey in 27 countries. Atherosclerosis.

[25] Pająk A., Szafraniec K., Polak M., Polakowska M., Kozela M., Piotrowski W., Kwaśniewska M., Podolecka E., Kozakiewicz K., Tykarski A. (2016). Changes in the prevalence, treatment, and control of hypercholesterolemia and other dyslipidemias over 10 years in Poland: The WOBASZ study. Pol. Arch. Med. Wewn..

[26] De Smedt D., de Bacquer D., de Sutter J., Dallongeville J., Gevaert S., de Backer G., Bruthans J., Kotseva K., Reiner Ž., Tokgözoğlu L. (2016). The gender gap in risk factor control: Effects of age and education on the control of cardiovascular risk factors in male and female coronary patients. The EUROASPIRE IV study by the European Society of Cardiology. Int. J. Cardiol..

[27] Dallongevillle J., De Bacquer D., Heidrich J., De Backer G., Prugger C., Kotseva K., Montaye M., Amouyel P., On Behalf Of The Euroaspire Study Group (2010). Gender differences in the implementation of cardiovascular prevention measures after an acute coronary event. Heart.

[28] Dodhia H., Kun L., Ellis H.L., Crompton J., Wierzbicki A.S., Williams H., Hodgkinson A., Balazs J. (2015). Evaluating quality and its determinants in lipid control for secondary prevention of heart disease and stroke in primary care: A study in an inner London Borough. BMJ Open.

[29] Zhao M., Vaartjes I., Graham I., Grobbee D., Spiering W., Klipstein-Grobusch K., Woodward M., Peters S.A. (2017). Sex differences in risk factor management of coronary heart disease across three regions. Heart.

[30] Silverman M.G., Ference B.A., Im K., Wiviott S.D., Giugliano R.P., Grundy S.M., Braunwald E., Sabatine M.S. (2016). Association Between Lowering LDL-C and Cardiovascular Risk Reduction Among Different Therapeutic Interventions: A Systematic Review and Meta-analysis. JAMA.

[31] Ference B.A., Ginsberg H.N., Graham I., Ray K.K., Packard C.J., Bruckert E., Hegele R.A., Krauss R.M., Raal F.J., Schunkert H. (2017). Low-density lipoproteins cause atherosclerotic cardiovascular disease. 1. Evidence from genetic, epidemiologic, and clinical studies. A consensus statement from the European Atherosclerosis Society Consensus Panel. Eur. Heart J..

[32] McCormack T., Dent R., Blagden M. (2016). Very low LDL-C levels may safely provide additional clinical cardiovascular benefit: The evidence to date. Int. J. Clin. Pract..

[33] Szymański F.M., Barylski M., Cybulska B., Wożakowska-Kapłon B., Krasiński Z., Mamcarz A., Widecka K., Płatek A.E., Dudek D., Mickiewicz A. (2018). Recommendation for the management of dyslipidemia in Poland — Third Declaration of Sopot. Interdisciplinary Expert Position Statement endorsed by the Polish Cardiac Society Working Group on Cardiovascular Pharmacotherapy. Cardiol. J..

[34] Mach F., Baigent C., Catapano A.L., Koskinas K.C., Casula M., Badimon L., Chapman M.J., De Backer G.G., Delgado V., Ference B.A. (2020). 2019 ESC/EAS Guidelines for the management of dyslipidaemias: *lipid modification to reduce cardiovascular risk*: The Task Force for the management of dyslipidaemias of the European Society of Cardiology (ESC) and European Atherosclerosis Society (EAS). Eur. Heart J..

[35] Zhao M., Woodward M., Vaartjes I., Millett E.R.C., Klipstein-Grobusch K., Hyun K., Carcel C., Peters S.A.E. (2020). Sex Differences in Cardiovascular Medication Prescription in Primary Care: A Systematic Review and Meta-Analysis. J. Am. Heart Assoc..

[36] Peters S.A., Colantonio L.D., Zhao H., Bittner V., Dai Y., Farkouh M.E., Monda K.L., Safford M.M., Muntner P., Woodward M. (2018). Sex Differences in High-Intensity Statin Use Following Myocardial Infarction in the United States. J. Am. Coll. Cardiol..

[37] Bradley C.K., Wang T.Y., Li S., Robinson J.G., Roger V.L., Goldberg A.C., Virani S.S., Louie M.J., Lee L.V., Peterson E.D. (2019). Patient-Reported Reasons for Declining or Discontinuing Statin Therapy: Insights From the PALM Registry. J. Am. Heart Assoc..

[38] Nanna M.G., Wang T.Y., Xiang Q., Goldberg A.C., Robinson J.G., Roger V.L., Virani S.S., Wilson P.W., Louie M.J., Koren A. (2019). Sex Differences in the Use of Statins in Community Practice. Circ. Cardiovasc. Qual. Outcomes.

[39] Rosano G.M., Lewis B., Agewall S., Wassmann S., Vitale C., Schmidt H., Drexel H., Patak A., Torp-Pedersen C., Kjeldsen K.P. (2015). Gender differences in the effect of cardiovascular drugs: A position document of the Working Group on Pharmacology and Drug Therapy of the ESC: Figure 1. Eur. Heart J..

[40] Tamargo J., Rosano G., Walther T., Duarte J., Niessner A., Kaski J.C., Ceconi C., Drexel H., Kjeldsen K., Savarese G. (2017). Gender differences in the effects of cardiovascular drugs. Eur. Heart J. Cardiovasc. Pharmacother..

